# Exploring genome characteristics and sequence quality without a reference

**DOI:** 10.1093/bioinformatics/btu023

**Published:** 2014-01-17

**Authors:** Jared T. Simpson

**Affiliations:** Ontario Institute for Cancer Research, Toronto, Canada

## Abstract

**Motivation:** The *de novo* assembly of large, complex genomes is a significant challenge with currently available DNA sequencing technology. While many *de novo* assembly software packages are available, comparatively little attention has been paid to assisting the user with the assembly.

**Results:** This article addresses the practical aspects of *de novo* assembly by introducing new ways to perform quality assessment on a collection of sequence reads. The software implementation calculates per-base error rates, paired-end fragment-size distributions and coverage metrics in the absence of a reference genome. Additionally, the software will estimate characteristics of the sequenced genome, such as repeat content and heterozygosity that are key determinants of assembly difficulty.

**Availability:** The software described is freely available online (https://github.com/jts/sga) and open source under the GNU Public License.

**Contact:** jared.simpson@oicr.on.ca

**Supplementary Information:** Supplementary data are available at *Bioinformatics* online.

## 1 INTRODUCTION

The availability of inexpensive DNA-sequence data has led to a vast increase in the number of genome projects. For example, the Genome10K project (Genome 10 K Community of Scientists, 2009) aims to sequence 10 000 vertebrate genomes in the upcoming years. Despite the advances in the production of DNA-sequence data, performing *de novo* assembly remains a significant challenge. This challenge was highlighted by the recent Assemblathon2 project (Bradnam *et al.*, 2013). In this competition sequence data was obtained for three vertebrate genomes. Twenty-one teams contributed assemblies of the three genomes, producing 43 assemblies in total. The quality of the assemblies was highly variable both between submissions for the same genome and within individual software packages across the three species. In our view, this variability stems from the practical difficulty of designing an assembly strategy (for instance, selecting software and its parameters) when little is known about the structure of the underlying genome and the quality of the available data. This article aims to address this uncertainty.

Most current genome assemblers are based on constructing a graph representing the relationship between sequence reads or their subsequences. The sequence of the underlying genome is modeled as a walk (or a set of walks) through the graph. The properties of the sequenced genome and quality of the input data is reflected by the structure of the graph; repeats, sequence variation (in a diploid or polyploid genome) and sequencing errors cause branches in the graph. These branches increase the difficulty of the assembly by obscuring the true walks that represent the sequence of the genome with many false alternatives. Below, we will show how we can estimate the individual contribution of sequence variants, repeats and sequencing errors to the branching structure of an assembly graph and we will discuss how the branching structure impacts assembly difficulty. Additionally, we will develop methods to perform rich quality assessment without a reference genome, complementing previously developed approaches (FastQC, http://www.bioinformatics.babraham.ac.uk/projects/fastqc/) (Keegan *et al.*, 2012; Schröder *et al.*, 2010; Wang *et al.*, 2012) by estimating sequence coverage, per-base error rates, insert size distributions and providing a visual method to assess coverage biases due to sequence composition (Dohm *et al.*, 2008; Ross *et al.*, 2013).

Our software is open source under the GNU Public License (version 3) and freely available online (https://github.com/jts/sga). The implementation uses the FM-index data structure, which allows queries to be performed over a large text collection while limiting memory usage. This framework allows our analysis pipeline to be run on 170 GB of human genome data in 24 h using 56 GB of memory on a single multi-core computer. The output of our software is a PDF report that allows the properties of the genome and data quality to be visually explored. By providing more information to the user at the start of an assembly project, this software will help increase awareness of the factors that make a given assembly easy or difficult, assist in the selection of software and parameters and help to troubleshoot an assembly if it runs into problems.

## 2 APPROACH

To efficiently calculate read-level metrics, we calculate each metric on a random subset of reads from the input sequence collection. For example, to estimate the per-base sequencing error rate, we sample a read from the entire read collection, use the FM-index to find reads that it overlaps, then count the number of mismatches in the overlapping set. Other read level metrics, such as the quality score distribution and fragment size distribution, are calculated with a similar sampling-based framework. By sampling reads we are able to estimate the metrics of interest without testing the full dataset. For each metric the number of trials is selected to minimize runtime while providing enough samples to generate meaningful results.

**Fig. 1.**
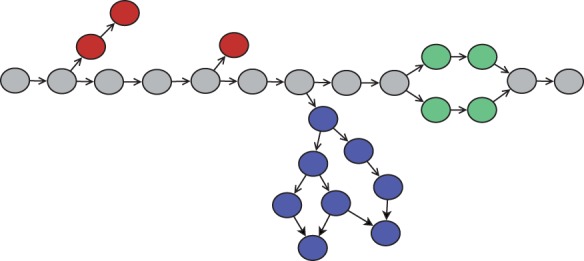
Schematic representation of a portion of a de Bruijn graph. Red vertices are *k*-mers-containing sequence errors. Green vertices are *k*-mers with sequence variation. Blue vertices are repeats

To quantify the structure of the de Bruijn graph, we sample *k*-mers and explore the local structure of the graph around the sampled vertex. Consider the schematic diagram of part of a de Bruijn graph in Figure 1.

A typical de Bruijn graph-based assembler will try to find and remove branches that are caused by sequencing errors (red vertices), find and collapse ‘bubbles’ caused by sequence variation (green vertices) and attempt to resolve branches caused by repeats (blue vertices). The rate at which the graph branches due to repeats and variation are key determinants of assembly difficulty. In Section 3, we design an algorithm to estimate how often such branches occur.

## 3 METHODS

### 3.1 Framework

The basic building block of the following methods is simply counting the number of times a particular string occurs in the read collection. For this task, we use the FM-index (Ferragina and Manzini, 2000) which allows the number of occurrences of a pattern *P* in the read collection to be counted in time proportional to the length of *P*. We will use the notation 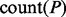 to refer to this procedure. As our dataset consists of DNA and we will often want to know the count of *P* and its reverse-complement, we define the function

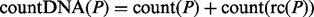

where the 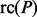 function returns the reverse-complement of *P*.

A second building block of our algorithms is sampling a read at random from the FM-index of the read collection *S*. We adapted the well-known functions to efficiently extract arbitrary substrings of the text from the FM-index (Ferragina *et al.*, 2004) to the restricted case of extracting an entire read from *S*. We will call the procedure to extract read *i* from the index 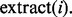 For a read collection with *n* reads, our sampling procedure simply draws a random number *i* from 0 to *n* – 1 then runs 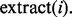


We can also use the FM-index to implicitly represent the structure of a de Bruijn graph. In Pevzner’s original definition of a de Bruijn graph *k*-mer subsequences of the reads are vertices in the graph (Pevzner *et al.*, 2001). Two vertices *X* and *Y* are connected by an edge if some read contains a (*k* + 1)-mer that contains *X* as a prefix and *Y* as a suffix, or vice-versa. This condition allows one to formulate the assembly as a tour of the graph that visits each edge at least once. As we do not require this condition for this work, we adopt the slightly simpler definition of the graph where the vertex set is the set of *k*-mer subsequences and the edges are defined by *k* – 1 overlaps between *k*-mers (Pell *et al.*, 2012; Simpson *et al.*, 2009). For our purposes, we consider a *k*-mer and its reverse complement to be the same vertex.

This definition of the graph allows us to determine the structure of the graph by simply performing *k*-mer count queries on the FM-index. Given a vertex sequence *X*, we can use the following procedure to find the neighbors of *X*. Write *X* as *X* = *aZ* where *Z* is the *k* – 1 suffix of *X*. We can then run 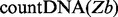 for 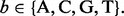 The *k*-mers with non-zero count represent the ‘suffix neighbors’ of *X*. The ‘prefix neighbors’ of *X* can be found similarly.

If a vertex has multiple suffix neighbors, we call it a ‘suffix branch’ (respectively, ‘prefix branch’).

### 3.2 The *k*-mer count distribution

The number of times a given *k*-mer occurs in the sequence reads depends on the *k*-mer’s genomic copy number, whether it contains a sequencing error, and the total number of *k*-mers drawn from the genome. To model *k*-mer counts, we assume a diploid genome and consider different types of *k*-mers. First, *k*-mers containing sequencing errors will occur at some rate 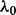 that depends on sequence quality and the total number of *k*-mers. Second, *k*-mers that are present on one of two parental chromosomes will occur at some rate 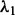 which is half the rate of *k*-mers that are present on both parental chromosomes, 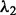. We will refer to these *k*-mers as heterozygous and homozygous, respectively. The *k*-mers that are repeated in the genome occur at rate 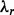 for 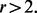 A natural generative model for the probability of observing a *k*-mer *c_i_* times for one component is a Poisson distribution (Lander and Waterman, 1988). In our case as we do not observe *k*-mers with count 0, we use a zero-truncated Poisson distribution:
(1)
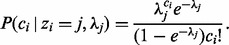



The latent variables *z_i_* indicate the type of the *k*-mer. For example 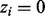 indicates *k*-mers that contain a sequencing error, 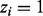 indicate heterozygous *k*-mers and so on. The probability of observing *c_i_* reads for *k*-mer *i* can be found by marginalizing out *z_i_*:
(2)
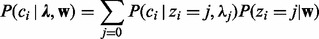

where 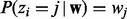 are the mixture proportions.

To fit the parameters of this model, we first construct an empirical distribution of *k*-mer counts by sampling 50 000 reads from the FM-index. Let 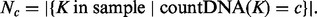 Intuitively, *N_c_* is the number of sampled *k*-mers with count *c* across all reads.

We initialize the model by calculating the mean count of homozygous *k*-mers *λ* as described in the supplement, fixing the number of components *n* to 10, setting 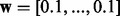 and 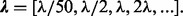 The vector **w** and 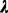 are iteratively updated using the Expectation-Maximization algorithm (Dempster *et al.*, 1977) until convergence or 30 iterations to obtain posterior estimates 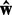 and 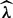. When updating 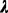 only 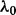 is changed. The parameters 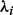 for 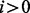 are fixed to constrain the model based on the assumption that the *k*-mer counts for each genomic copy-number state are directly determined by overall sequence coverage.

In principle, it is preferable to model the counts as a mixture of negative binomial distributions to model over dispersion of the count data. Chikhi and Medvedev (2013) recently modeled the count distribution using a mixture of Gaussians for this purpose. We found the Poisson mixture was sufficient for the following applications so opted for the simpler model.

### 3.3 Estimating genome size

It is useful to know the expected genome size to evaluate the completeness of the final assembly. Previously, genome size has been estimated from the distribution of *k*-mer counts (Li *et al.*, 2009). Here we adapt this method by explicitly correcting for sequencing errors.

Assuming all reads are length *l* and the reads do not contain sequencing errors, there is a simple relationship between *k*, the number of reads, *n*, and genome size, *G*. There are 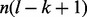 *k*-mers in the reads and 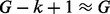 (as 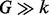) *k*-mers in the genome. The mean number of times a homozygous *k*-mer appears in the reads is therefore:
(3)
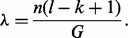



If we know *λ*, which we approximate from the count distribution as described in the previous section, *G* can easily be calculated. If the reads contain sequencing errors, this calculation requires modification. In this case, the quantity 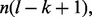 the total number of *k*-mers in the reads, is a mixture of genomic *k*-mers and artificial *k*-mers containing errors. However, *λ* indicates the mean count of homozygous *k*-mers only, and does not include *k*-mers containing errors. Therefore the calculation 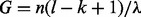 will overestimate *G* as 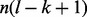 is inflated by *k*-mers with errors. We correct for this effect using *w*_0_, the proportion of *k*-mers that contain errors from our mixture model. Our genome size calculation therefore becomes:
(4)
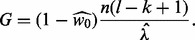



We use *k* = 31 when estimating genome size.

### 3.4 Branch classification

Sequencing errors, sequence variants and repeats cause branches in the de Bruijn graph contributing to assembly difficulty (Fig. 1). We can estimate the rate of branching in the graph that can be attributed to each of these branch types by coupling a graph traversal with a probabilistic classifier. For this analysis, we assume that the sequenced genome is diploid. We start by sampling a read from the FM-index then iterating over all *k*-mers in the read. We check each *k*-mer that satisfies 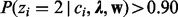 (the posterior probability of the *k*-mer being homozygous) for a suffix branch. To minimize the impact of systematic errors (Guo *et al.*, 2012), we require that a neighboring *k*-mer is seen on both sequencing strands to be a valid edge in the graph. If *k_i_* has more than one suffix neighbor, we attempt to classify the branch. Let *k_a_* and *k_b_* be the two highest coverage neighbors of *k_i_* with counts *c_a_* and *c_b_*. We set *k_a_* to be the higher coverage neighbor 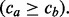


We classify each such branch using a modified version of the probabilistic model designed by Iqbal *et al.* (2012). Initially we modeled the total coverage of the branch, 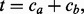 using a Poisson distribution with mean *λ* under the variant and error models and 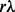 in the repeat model (for 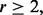 representing the repeat copy number). This model tended to misclassify repeats as variants in the case when *k_i_* is from a low-coverage region of the genome, as *t* is correlated to *c_i_* and therefore undersampling *k_i_* -biased *t* to be smaller than expected. To account for this, we define a new variable without the dependency on *c_i_*. Let *c_ia_* (respectively, *c_ib_*) be the number of reads that contain both *k_i_* and *k_a_* (*k_i_* and *k_b_*). We define 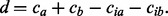 Intuitively, *d* is the number of reads that contains *k_a_* or *k_b_* but not *k_i_*. Under the variant and error model this is only possible when *k_a_* or *k_b_* is the first *k*-mer of a read or if there is a sequencing error in the first base of *k_i_*. Both of these cases are relatively rare so *d* is expected to be very small under the variant and error model. Under the repeat model *k_a_* and *k_b_* appear in more genomic locations than *k_i_*. This gives more opportunities to sample *k*-mers covering *k_a_* and *k_b_* so we expect *d* to be relatively large.

We use the following distributions for *d*, conditional on the branch classification:
(5a)
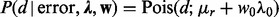

(5b)
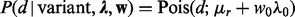

(5c)
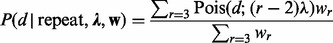

where 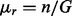 is the density of read-starting positions along the genome. In the repeat model, we sum over repeat states of the mixture model, where each state represents an integral number of extra genomic copies of *k_a_* and *k_b_*.

The second source of information is the coverage balance between *k_a_* and *k_b_*. If *k_a_* and *k_b_* represent a variant, we expect each *k*-mer to be equally well represented. If the branch is due to an error we expect most reads to support the higher coverage neighbor, *k_a_*. We model coverage balance with the following distributions:
(6a)
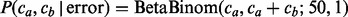

(6b)
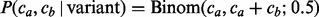

(6c)
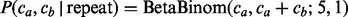



Here 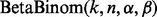 is the probability mass function of the Beta-Binomial distribution parameterized by α and β and 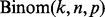 is the probability mass function of the Binomial distribution parameterized by *p*. The parameters α and β of the Beta-Binomial under the repeat model are chosen to reflect our uncertainty of the genomic copy-number configuration of *k_a_* and *k_b_* (Iqbal *et al.*, 2012).

We calculate the posterior probability of each classification using Bayes’ rule assuming independence of *d* and 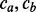 and a uniform prior on the classifications. We then estimate branch rates from the classifications. We count the number of branches classified as each type (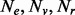) and the number of homozygous *k*-mers that were checked for a branch (*N_h_*). Rather than classifying each branch to the type with the largest posterior probability, we use soft classifications and update the branch counts with the expectation from the posterior of the model:
(7a)
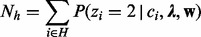

(7b)
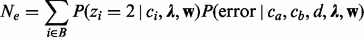

(7c)
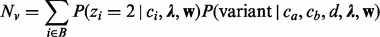

(7d)
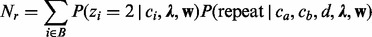



Here *H* is the set of sampled *k*-mers that were checked for a branch and *B* is the subset of *H* consisting of *k*-mers that have a suffix branch.

We perform this classification on every *k*-mer in 1 000 000 randomly sampled reads for *k* 21–71 in increments of 5. For the output plots in the PDF report the branch rates are calculated as 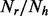 and 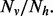 If the number of branches for a classification is <2, no point is plotted for that value of *k*.

This model has limited power to distinguish between sequence errors and variants when *λ* is small. Additionally, if *λ* is too small we will simply not observe variant branches in the graph due to both alleles not being represented in the sequence data. Therefore, we do not output classifications when 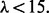


### 3.5 Estimating position-specific error rates

Sequencing errors complicate de Bruijn graph assembly by lowering effective *k*-mer coverage and adding false branches to the graph. In overlap-based assembly sequencing errors must be accounted for by either allowing the overlaps to have mismatches or gaps, which leads to false-positive edges in the graph, or by error correcting the reads prior to graph construction (Simpson and Durbin, 2012).

To estimate the sequencing-error rate as a function of base position within the read, we compute read–read overlaps that are seeded by short exact matches. We begin by sampling a read *R* from the FM-index and computing the set of reads that share a 31-mer with *R*. For each read in this candidate set we compute an overlap between the read and *R*. To avoid spurious matches between repetitive sequences, we require the overlap is at least 50 bp in length and the percent identity is at least 95%. We construct a multiple alignment using *R* and the pair-wise overlaps for reads meeting this threshold. We then compute a consensus sequence for each column of the multiple alignment. A base call 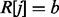 is considered to be incorrect if *b* does not match the consensus base, at least three reads support the consensus base and fewer than four reads support base call *b*. We calculate the error rate at position *j* as:
(8)
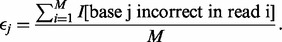



We use *M* = 100 000 for the figures in this manuscript. To avoid excessively long computation time for repetitive reads, we skip 31-mers that are seen >200 times in the reads when computing the candidate overlap set.

### 3.6 Estimating the fragment-size distribution

To help resolve long genomic repeats, read pairs are typically obtained by sequencing both ends of a DNA fragment. The fragment size range is determined during preparation of the DNA prior to sequencing. To ensure that the sequenced fragment size distribution matches the expected distribution, we developed a method to estimate the fragment size distribution. We begin by sampling a read pair, *X* and *Y*, from the FM-index. Starting from the first 51-mer of *X*, we perform a greedy search of the 51-mer de Bruijn graph by choosing the highest coverage branch as the next vertex in the search. The search stops when the first 51-mer of *Y* is found, there are no possible extensions or 1500 iterations have passed. If a complete walk from *X* to *Y* is found, the length of the walk in nucleotides is emitted as the fragment size. If sequence coverage is low, this method of estimating the fragment size distribution may be biased towards shorter fragments, as it is more likely that a walk representing a long fragment is broken by lack of coverage. For Section 4.7, 100 000 read pairs were sampled.

### 3.7 Simulating de Bruijn assembly

We designed a method to simulate the output of a de Bruijn assembler to allow the dependency between *k*-mer size and contig size to be explored. For small *k* the graph will branch more often due to repeats than for large *k* but for large *k* we are less likely to sample the complete set of genomic *k*-mers leading to coverage breaks. Our simulation allows the balance between these factors to be explored by performing walks through a de Bruijn graph mimicking the performance of an assembler that is able to identify and resolve false branches that are caused by errors and bubbles that are caused by variants. As opposed to most assemblers which classify branches as errors, variants or repeats based the topology of the graph we use the probabilistic model developed in Section 3.4 to guide the graph traversal.

We begin by sampling a read at random and calculating the probability that the first *k*-mer of the read is a homozygous *k*-mer as in the branch classification method. If the probability is <0.50, we discard this read and start again. Otherwise we begin a new contig starting from the first *k*-mer of the read.

Let *X* be the current *k*-mer of the contig. We check *X* for a branch as in our branch classification method. If *X* does not have a branch, or has a branch that is classified as an error or variant, we iterate from the highest-coverage neighbor. If *X* does not have a neighbor or has a repeat branch we terminate extension of the contig. This procedure occurs for both the suffix neighbors of the initial *k*-mer and the prefix neighbors. Once the extension has terminated in both directions the number of *k*-mers visited is written to the output file.

To avoid excessively long computation time we cap the maximum walk length at 50 000 and stop extension if a particular *k*-mer is visited twice. We also do not allow a given walk to be used multiple times by recording all visited *k*-mers in a bloom filter. Starting *k*-mers that are present in the bloom filter are skipped. We perform 20 000 walks for each *k* from 21 to 91 in increments of 5.

### 3.8 Computations

The program to calculate the genome characteristics and qc metrics is implemented as a module of the SGA assembler in C++. This program writes the results to a JSON file, which is read by a Python script to generate the PDF report. The computations performed in this article are fully reproducible by downloading and running the following Makefile:

https://github.com/jts/preqc-paper/tree/master/bin/generate_data.make

The Makefile will download the input data from public repositories, run SGA, and then generate the final reports. Version 0.10.12 of SGA was used to generate the data and figures for this article. The JSON-formatted results are available online (ftp://ftp.sanger.ac.uk/pub/js18/preqc-paper).

The computation time for the human data, the largest set used in the article, was 13 h (elapsed time) to download the data, 18 h to build the FM-index and 6 h to calculate the metrics. The memory high-water mark was 56 GB during construction of the FM-index. For the other genomes the index construction time ranged from 1 h for the yeast data to 14 h for the snake data. The metrics calculation runtimes ranged from 2 h (yeast) to 9 h (bird).

## 4 RESULTS

### 4.1 Input data

In the following sections, we demonstrate the output of our program using freely available data from genomes of varying complexity. The selected datasets and their accessions are:

- *Saccharomyces cerevisiae* (ERR049929),
- *Melopsittacus undulatus*, a budgerigar from Assemblathon2 (ERR244146),
- *Maylandia zebra*, a Lake Malawi Cichlid from Assemblathon2 (SRX033046),
- *Boa constrictor constrictor*, a snake from Assemblathon2 (ERR234359-ERR234374),
- *Crassostrea gigas*, a Pacific oyster (SRR322874-SRR322877),
- *Homo sapiens*, a human genome (ERR091571-ERR091574).

For simplicity and consistency with the Assemblathon2 paper, we will refer to these datasets as ‘yeast’, ‘bird’, ‘fish’ ‘snake’, ‘oyster’ and ‘human’. The yeast genome was selected to provide an example of an uncomplicated genome that is typically straightforward to assemble. In contrast, the oyster genome is highly heterozygous and repeat-rich. This genome was recently sequenced using a fosmid-pooling strategy after whole genome assembly failed to produce satisfactory results (Zhang *et al.*, 2012). The human and Assemblathon2 datasets represent a range of large eukaryotic genomes of varying heterozygosity and repeat content. Multiple high-coverage sequencing libraries are available for the human and Assemblathon2 samples. For each genome a single library was selected for analysis. For the oyster data all three short-insert libraries are used for the inbred sample to provide adequate coverage to infer the properties of the genome. The yeast dataset was downsampled from 500X coverage to 40X to be consistent with the other datasets. We first describe our estimates of genome characteristics, followed by our data quality metrics.

### 4.2 Exploring heterozygosity

**Fig. 2.**
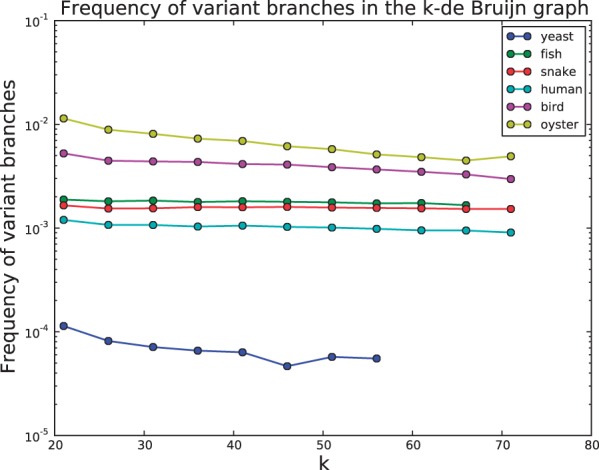
The estimated variation branch rate for each genome as a function of *k*. Branch rate estimates for the yeast and fish data are not available for all *k* due to insufficient coverage

Allelic differences in a diploid or polyploid genome generate branches in an assembly graph with the characteristic ‘bubble’ structure (Zerbino and Birney, 2008) shown in Figure 1. Most graph-based assemblers have functions to search for these structures in the graph and remove them. While these algorithms are typically effective at removing isolated allelic differences, high density variation can make assembly challenging (Donmez and Brudno, 2011; The Potato Genome Sequencing Consortium, 2011; Zhang *et al.*, 2012). We used the branch classifier developed in Section 3.4 to estimate the sequence variant branch rate in our test datasets. Figure 2 depicts the rate of variant branches in a de Bruijn graph as a function of *k*. Approximately 1 in 1000 vertices in the de Bruijn graph of the human sample has a variant-induced branch, which is consistent with the rate of heterozygous variation found by reference-based analysis of this genome (supplemental results).

It is easy to see from Figure 2 that the oyster genome has the highest density of variant branches, indicating the genome is highly heterozygous. As observed in Zhang *et al.* (2012) this extreme heterozygosity makes assembly significantly challenging. Of the three Assemblathon genomes, the bird genome has the highest heterozygosity while the fish and snake datasets had similar estimated heterozygosity. The human genome contains the least level of variation within the diploid species.

A low level of branching in the yeast dataset is attributed to sequence variation (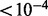 branch rate). As the sequenced yeast was haploid these likely represent misclassification of systematic sequencing errors or repeats.

### 4.3 Exploring genome repeat content

Genomic repeats also cause branches in the assembly graph. As repeat branches tend to be difficult to resolve, often requiring long-range paired-end data to jump over the repetitive region (Weber and Myers, 1997) the number of repeat-induced branches is a key indicator of assembly difficulty (Kingsford *et al.*, 2010).

**Fig. 3.**
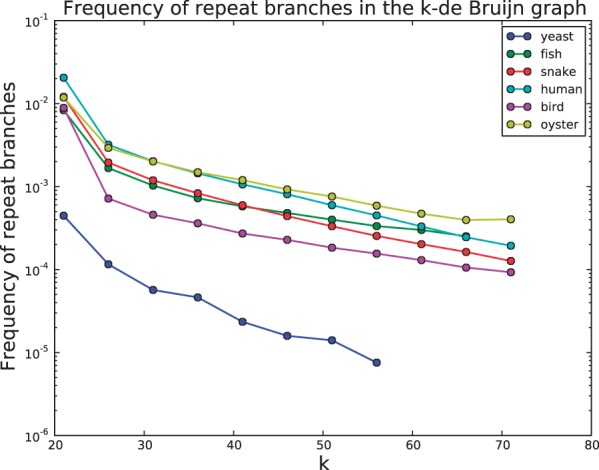
The estimated repeat branch rate for each genome as a function of *k*

We use the output of our classifier to estimate the rate at which repeat-induced branches appear in a de Bruijn graph as a function of *k* (Fig. 3). As expected, the rate of repeat-induced branches clearly decreases as a function of *k* for all datasets. The difficulty of assembling the oyster genome is again reflected in this analysis. For 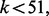 the oyster graph has a comparable repeat branch rate to the human graph despite oyster’s much smaller genome size. For larger *k*, the oyster graph has the highest branch rate of all datasets. Likewise, the fish genome is more repetitive than what might be expected from its relatively small genome size.

The yeast genome branches very infrequently due to repeats. Coupled with the lack of variation shown in the previous section, this suggests that even with small *k* the de Bruijn graph of the yeast data is relatively uncomplicated and should be straightforward to assemble.

### 4.4 Genome size estimates

The final genome characteristic that we estimate is the size of the genome itself. Table 1 presents a comparison of our genome size estimates to either the reference size or a recent published estimate.

**Table 1.** The genome size estimates from our method compared to previously published estimates

Genome	Estimate (MB)	Published size (MB)	Source
Yeast	13	12	Goffeau *et al.* (1996)
Oyster	549	545–637	Zhang *et al.* (2012)
Fish	889	1000	Bradnam *et al.* (2013)
Bird	1086	1200	Bradnam *et al.* (2013)
Snake	1467	1600	Bradnam *et al.* (2013)
Human	2895	3102	GRC37

### 4.5 Assessing genome coverage

To facilitate genome assembly the genome must be sequenced redundantly. The parameters key to the success of an assembly, particularly the overlap length or *k*-mer size in de Bruijn assembly, are tightly linked to the depth of coverage. If the parameters to the assembler are too stringent, for instance large *k* or long overlaps are requested, then the graph may become disconnected due to lack of coverage. Conversely, if the parameters are too permissive then the graph may contain an unacceptable number of repeat branches. The parameters are usually chosen (or learned from the data) to maximize stringency subject to available coverage.

**Fig. 4.**
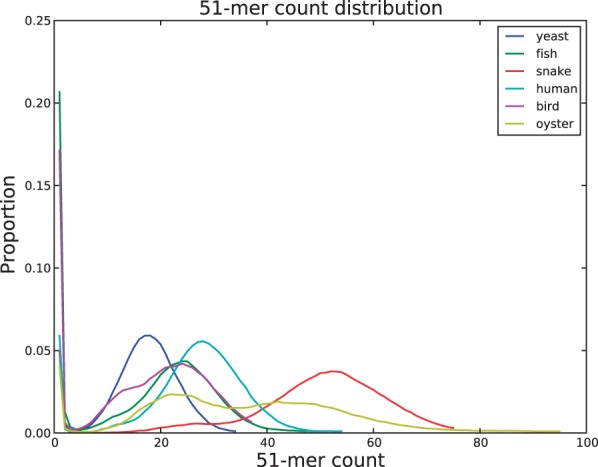
A histogram of 51-mer frequencies for each dataset

We have developed multiple methods to assess the coverage of a given dataset. The first method is a histogram of *k*-mer counts for a fixed *k* (by default *k* = 51). An example is shown in Figure 4.

On the *x*-axis are *k*-mer counts and the *y*-axis is how frequently *k*-mers seen *x* times occur in the sampled data. For example, 5–20% of *k*-mers are seen only once. These *k*-mers with low count typically contain sequencing errors (Kelley *et al.*, 2010; Pevzner *et al.*, 2001; Simpson and Durbin, 2012). The remaining *k*-mers, those with higher count (>5 occurrences), are typically error-free and form the substrate of the assembly graph. Ideally the error-free *k*-mers are well separated from *k*-mers containing errors to allow easy identification and correction of errors. The snake data is an excellent example of the desired separation, while the yeast data would benefit from more sequencing data or choosing a smaller *k*.

The count distribution also informs our understanding of heterozygosity. The oyster and bird data, which have the highest estimated heterozygosity by our branch-classification method, have two noticeable peaks in the distribution of error-free *k*-mers. One peak corresponds to *k*-mers present on both parental haplotypes (at count 46 for oyster, 24 for bird) and one peak for *k*-mers covering heterozygotes (22 for oyster, 13 for bird). The oyster heterozygosity is so high that the peak at count 22 is the mode of the error-free 51-mer distribution.

Sequence coverage is known to be dependent on the GC content of the sampled fragment (Ross *et al.*, 2013). For extremely biased genomes, it can be difficult to cover the entire genome with sequence reads (Kozarewa *et al.*, 2009). To visually assess coverage as a function of GC content, we generate a 2D histogram of (GC-content, *k*-mer count) pairs. If sequence coverage is independent of GC content then the distribution of sequence coverage within each column will be the same. As an example, the fish data has a relatively uniform coverage profile across therange of GC content (Supplementary Fig. S1A). The yeast data is skewed with higher GC sequences having lower coverage on average (Supplementary Fig. S1B). However, the overall coverage is high enough that this mild bias likely does notimpact the assembly. The heteroyzgosity of the oyster data is clearly visibile as two distinct clusters of *k*-mers (Supplementary Fig. S1C).

### 4.6 Simulating contig assembly

**Fig. 5.**
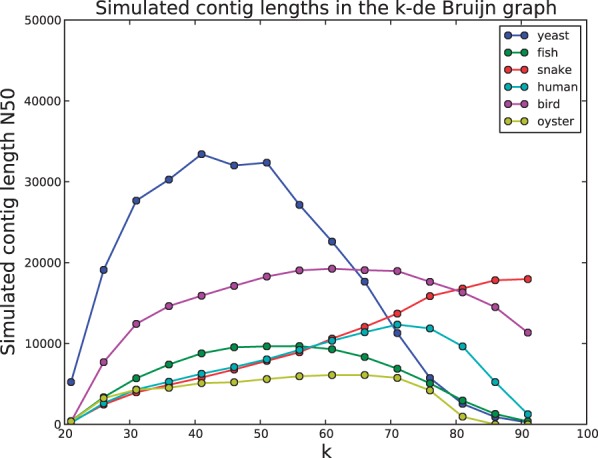
The N50 length of simulated contigs for *k* from 21 to 91, in increments of 5

In Section 3.7, we describe how our branch classifier can be used to simulate the output of a de Bruijn assembler. Figure 5 shows the simulated contig N50 as a function of *k* for the six test genomes. For most datasets, there is a value of *k* that maximizes N50 contig length by striking a balance between ability to resolve short repeats and ensuring the graph is well-connected. The yeast data is the best example of this with a peak at intermediate *k*. The snake data is able to support a very large *k* as the high-sequencing depth ensures the graph remains well-connected even for large *k*. By this assessment, the oyster data is again the most difficult to assemble.

### 4.7 Assessing error rates and insert sizes

**Fig. 6.**
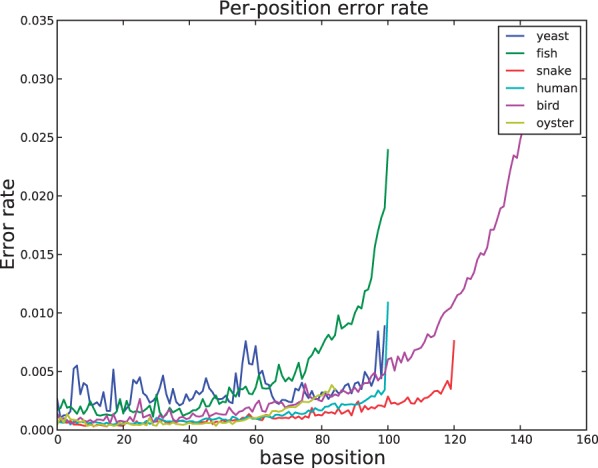
The per-base error rate for each dataset calculated by read–read overlaps

To infer per-base sequencing error rates, we calculate read-read overlaps and compare each read to the consensus sequence of reads it overlaps. All datasets show the tendency of higher error rate towards the 3′-end of the sequence read characteristic of Illumina data (Nakamura *et al.*, 2011) (Fig. 6). Most datasets have <1% error rate across the length of the read. The distribution of quality scores along the length of the reads shows a similar trend (Supplementary Fig. S2).

Paired-end sequence data is commonly used to help resolve repeats that are longer than the read length. To help ensure that the sequenced fragments match the expected size determined by the DNA library preparation, we infer the insert size distribution by performing walks through the assembly graph that begin and end on either end of a read pair (Supplementary Fig. S3). In this figure, the oyster data has three modes as it is a mixture of three separate paired-end libraries.

### 4.8 Model accuracy

Finally, we performed a simulation to test the accuracy of our branch classification model. We performed this assessment by obtaining a diploid reference genome for the human sample (see Supplemental Material). We directly calculated the variant and repeat branch rate from the de Bruijn graph of the diploid reference genome. We also simulated 40X coverage of this diploid reference and estimated branch rates using the same methodology as the real NA12878 data. We expect that the branch rate estimates from the simulated data should match the direct calculations from the reference graph. The branch rates for the real data should be close to those of the reference graph and simulated data but may differ slightly due to the way the diploid reference genome was prepared or biases in real data that our classification model does not account for.

In Supplementary Figure S4, the variant and repeat branch rates for the reference graph, simulated reads and real NA12878 data is shown. The estimated repeat branch rate for the simulated data and real data closely match the repeat rate of the diploid reference genome. The variant branch rate for the simulated data closely matches the reference calculation, except for very small *k*. At low *k* there is a very high density of repeat branches, which suggests misclassification of repeats may lead to an overestimation of the variant branch rate. The variant branch rate for the real dataset is consistently higher than the simulation and direct reference calculation. This difference may be due to misclassification of systematic sequencing errors as variants or indicate that an incomplete variant set was used to construct the diploid reference genome.

## 5 DISCUSSION

While the development of new genome assembly methods continues, comparatively little attention has been paid to assisting the user from a practical standpoint. Our program, along with tools like VelvetOptimiser (http://bioinformatics.net.au/software.velvetoptimiser.shtml) and KmerGenie (Chikhi and Medvedev, 2013), attempts to fill this gap. The program we developed helps the user perform quality checks on their data while simulatenously assessing the difficulty of the assembly by measuring the branching structure of a de Bruijn graph. By helping the user better understand their data, our program makes progress towards the goal of making assembly an easier and more consistent process. The ability to identify low quality data early in the assembly process will help to avoid effort wasted on unsuitable data, and guide the application of optional preprocessing steps like quality trimming the reads. The PDF report generated by the software provides a medium for discussing a given assembly problem, for instance when getting help on a mailing list or online.

The methods described in this article are our initial attempt at assessing data quality and genome characteristics. There are two promising ways to extend this work. First, we have limited our model to diploid genomes. Extending the model to higher ploidly would increase the range of genomes that could be characterized. Second, we only describe methods to assess short insert paired-end libraries. Many assembly projects use a range of paired end libraries including multi-kilobase insert sizes. The addition of methods to perform quality controls on these libraries would be a valuable addition to our program.

## Supplementary Material

Supplementary Data
