# Supplementary material for: Exploring genome characteristics and sequence quality without a reference
Source: Bioinformatics. 2014 Jan 17;30(9):1228–35. doi: 10.1093/bioinformatics/btu023 (PMC3998141; doi:10.1093/bioinformatics/btu023)
Supplement: Supplementary Data [file supp_30_9_1228__index.html]

Exploring genome characteristics and sequence quality without a reference — Exploring genome characteristics and sequence quality without a reference — Supplementary Data 

# Exploring genome characteristics and sequence quality without a reference

## Supplementary Data

files

**Files in this Data Supplement:**

- Supplementary Data - pdf file
