# Supplementary material for: Exploring genome characteristics and sequence quality without a reference
Source: Bioinformatics. 2014 Jan 17;30(9):1228–35. doi: 10.1093/bioinformatics/btu023 (PMC3998141; doi:10.1093/bioinformatics/btu023)
Supplement: Supplementary Data [file supp_btu023_btu023supplement.pdf]

Exploring Genome Characteristics and Sequence Quality Without a Reference
Supplementary Methods and Figures

Supplemental Methods

Fitting the mean depth of homozygous k-mers

In section 3.2 we describe a mixture model for k-mer counts. The first part of the model fitting procedure is to estimate λ, the mean count of homozygous k-mers. We do this by examining the empirical k-mer count distribution (figure 4 in the main text). Provided k is large enough that most k-mers are not repetitive, the genome is not very heterozygous and the sequencing error rate is low, the majority of k-mers will be homozygous and the mode of the distribution provides an estimate of λ. In our experience most data sets meet these assumptions; we handle the exceptions as follows. For data with a high error rate, k-mers seen a single time ($c = 1$) may be the most frequent. To avoid selecting the mode corresponding to erroneous k-mers, we select the mode after the first local minimum of the distribution. For highly heterozygous data the mode of the distribution may correspond to heterozygous k-mers (for example the oyster data in figure 4). To account for this we explicitly check for a secondary peak. Let m be the mode of the distribution, which has height N_m. If there is a second peak at $2m$ with height $N_{2m} \geq N_m/2$ we use $2m$ as our estimate of λ instead of m.

GC vs coverage plots

The plots in supplemental figure 1 are two-dimensional histograms of (GC, count) pairs. To calculate the input data, we sample $100,000$ reads from FM-index and run `countDNA` on the first 31-mer of the read. If the count is one, we reject the read as the first 31-mer likely represents an error. Otherwise, we calculate the proportion of GC bases in the entire read and emit a (GC, count) pair. These pairs are input into the `histogram2d` function of the numpy python library (`http://www.numpy.org/`).

Calculating per-base quality scores

The average quality score per base position is calculated by sampling every 20th read in the input FASTQ file up to a maximum of 10,000,000 sampled reads.

Branch classification accuracy assessment

To assess the accuracy of our branch classifier, a diploid reference genome for NA12878 was downloaded[1] . This reference genome was constructed from SNP and indel calls for NA12878 phased onto parental chromosomes [1]. This reference genome was processed to change uncalled bases to a random base. From this diploid reference genome, we simulated sequence reads using DWGSIM (`https://github.com/nh13/DWGSIM`) with the following command:

```
dwgsim -C 20 -r 0.0 -1 100 -2 100 -e 0.0001-0.005 -E 0.0001-0.005
-y 0 -d 300 -s 30 NA12878.diploid.fa prefix
```

The simulated data was processed using the same analysis pipeline as the real datasets.

Additionally, we calculated the variant and repeat branch rate from the de Bruijn graph of the diploid reference. The method is similar to section "Branch classification" above, except in the place of the probabilistic model we use simple counts to classify the structures in the graph. If a sampled k-mer has a count of exactly 2 (one copy on each parental chromosome), we consider it to be a homozygous k-mer. Branches are detected by finding homozygous k-mers that have multiple neighbors. When such a branch is found, we classified the branch as a variant if each neighbor had count 1 in the diploid reference, otherwise we called the branch a repeat.

References

[1] Joel Rozowsky, Alexej Abyzov, Jing Wang, Pedro Alves, Debasish Raha, Arif Harmanci, Jing Leng, Robert Bjornson, Yong Kong, Naoki Kitabayashi, Nitin Bhardwaj, Mark Rubin, Michael Snyder, and Mark Gerstein. Allele-Seq: analysis of allele-specific expression and binding in a network framework. *Molecular systems biology*, 7(1), August 2011.

Supplemental Figures

[1] `http://sv.gersteinlab.org/NA12878_diploid/NA12878_diploid_dec16.2012.zip`

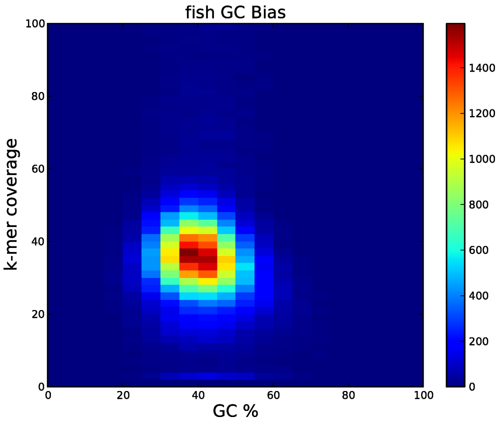

(a)

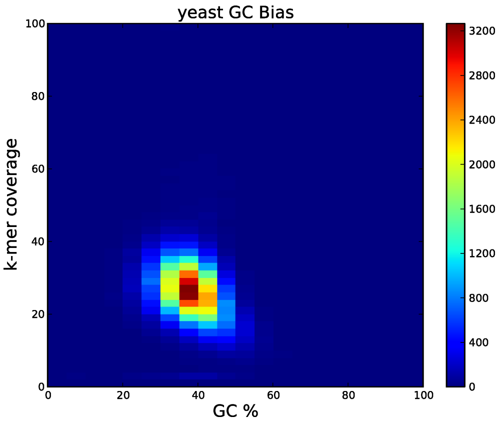

(b)

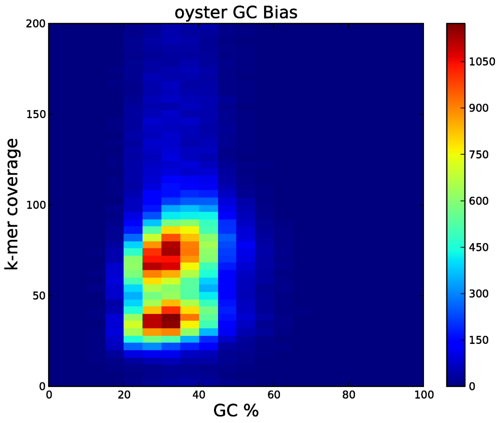

(c)

Figure 1: Two-dimensional histogram of (GC, count) pairs for 31-mers for the fish (a), yeast (b) and oyster (c) data sets

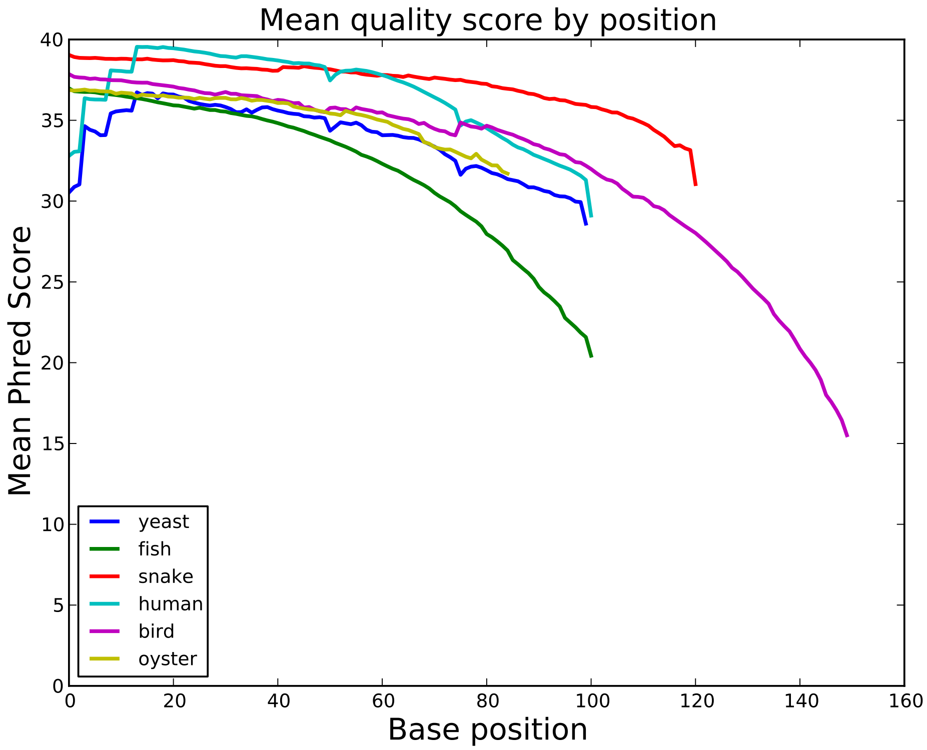

Figure 2: The mean quality score per base position for each data set

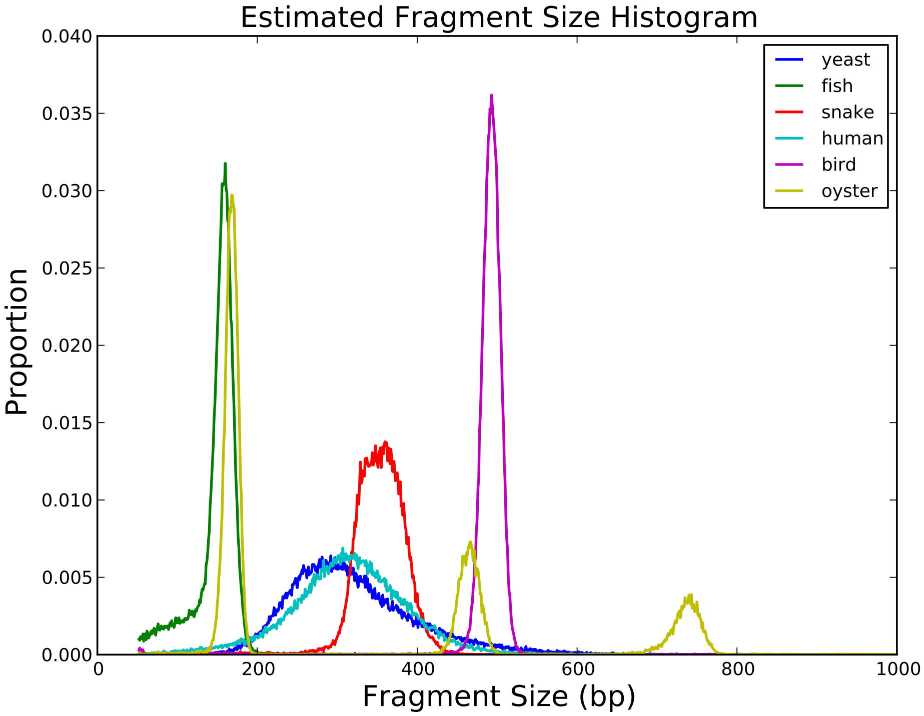

Figure 3: The estimated paired-end fragment size for each data set. The oyster data set is a mixture of three libraries.

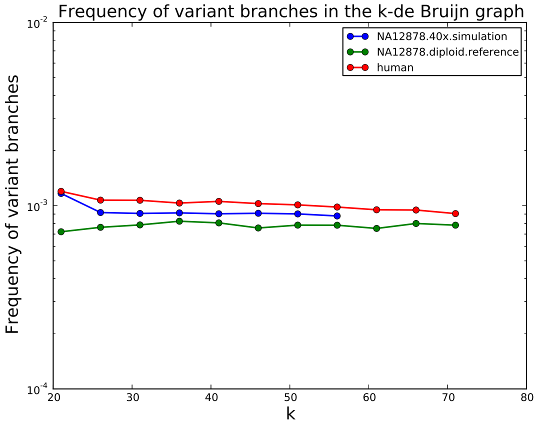

(a)

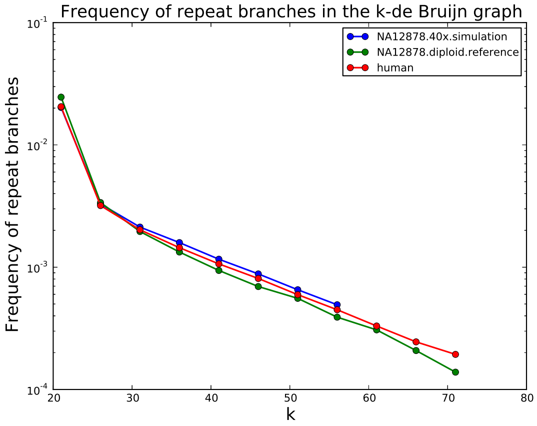

(b)

Figure 4: Variation (a) and repeat (b) branch rate estimated from real data, simulated data and the diploid reference genome of NA12878
